# The Effect of Synthetic Zeolite on the Curing Process and the Properties of the Natural Rubber-Based Composites

**DOI:** 10.3390/polym16223228

**Published:** 2024-11-20

**Authors:** Sonja Stojanov, Olga Govedarica, Marija Milanović, Julijana Žeravica, Berta Barta Hollo, Dragan Govedarica, Mirjana Jovičić

**Affiliations:** 1 Faculty of Technology Novi Sad, The University of Novi Sad, 21000 Novi Sad, Serbia; sonja.stojanov@uns.ac.rs (S.S.); oborota@uns.ac.rs (O.G.); majam@uns.ac.rs (M.M.); julijana.blagojevic@uns.ac.rs (J.Ž.); dragang@uns.ac.rs (D.G.); 2 Faculty of Sciences, The University of Novi Sad, 21000 Novi Sad, Serbia; hberta@uns.ac.rs

**Keywords:** natural rubber, zeolite, elastomeric materials

## Abstract

Zeolites, known for their unique structural and catalytic properties, are added to the natural rubber matrix to investigate their influence on the vulcanization process and the resultant properties of composites. The natural rubber-based composites were masticated with 4A synthetic zeolite (0, 5, 10, 15, 20, and 30 phr). The curing of the rubber compounds was monitored on a moving die rheometer at 150 °C. The isothermal DSC method was also used to study the curing process at 150 °C, 160 °C, and 170 °C. Based on the obtained results, it is assumed that there is an interaction between the components of the curing system and the surface of the zeolite particle, and that is why the vulcanization reaction starts earlier with an increase in zeolite in the rubber mixture. This underscores the significant role of zeolite in accelerating the curing reaction of natural rubber-based compounds. The composites were vulcanized in a press at 150 °C for 15 min. The chemical structure was analyzed using FTIR, and the sample morphology was examined using SEM. The degree of swelling in toluene and distilled water was determined. The tensile strength values, modulus of elasticity at 100% and 300% elongation, and elongation at break were measured using a universal testing machine. Hardness was assessed according to the Shore A scale. With a small addition of zeolite (up to 10 phr), there is no significant change in the tensile strength values. However, adding a considerable amount of zeolite to a natural rubber matrix results in a deterioration of the tested mechanical properties. It can be assumed that with large proportions of zeolite 4A MS in the composites, the mechanical properties deteriorated due to increased porosity. The amount of added zeolite affects the initial stages of thermal decomposition of the examined samples and the rest after the analysis at a temperature of 500 °C.

## 1. Introduction

Clays, especially kaolin (kaolinite) and zeolites, are the aluminosilicates most often used as fillers in natural rubber. These minerals are valued for enhancing mechanical properties, improving barrier properties, and reducing costs [1,2,3,4]. The kaolin surface contains abundant aluminum hydroxyl bonds, and there are strong van der Waals forces and hydrogen bonds between the lamellae of kaolin, making kaolin incompatible with non-polar natural rubber [5]. However, to achieve the required reinforcement specifications, kaolin must be modified to enhance their rubber-filler compatibilities [4]. Therefore, the surface chemical modification of kaolin is needed to improve the dispersion of kaolin in polymers [6]. Functionalized silica kaolinite mineral was well dispersed in the rubber and improved filler-rubber interactions, thus improving mechanical properties [1].

Zeolites are crystalline aluminosilicate minerals with a porous structure. Synthetic zeolites are engineered versions of these minerals. They have found wide applications in industrial processes related to adsorption, ion exchange, and catalysis [7]. They are also applied in the rubber industry due to their unique properties. Some typical applications of zeolites in the rubber industry include reinforcement of rubber compounds, reduction in rolling resistance, wear resistance improvement, moisture scavenging, improved thermal stability, controlled swelling, and dimensional stability [8,9,10].

When incorporated into a rubber matrix, zeolites can act as moisture scavengers, reducing the impact of water on rubber composites. This moisture-scavenging property can be advantageous in rubber compounds to protect against moisture-induced degradation or in applications where exposure to water may compromise the performance of rubber products (such as seals, gaskets, and other precision rubber components). At the same time, zeolite is a non-toxic and environmentally friendly product. It reduces the emission of unwanted substances that arise during production and prevents their negative effects on human health and the environment. The high absorption power of zeolite enables special characteristics of the end products, such as the absorption of odors and unwanted gases inside the product or in its surroundings.

The effectiveness of zeolite in improving these properties depends on the specific type of zeolite that is used, its content in the rubber compound, its particle size, and the overall rubber compound formulation. The interaction between zeolite and natural rubber is complex, and carefully tailoring formulations is necessary to achieve the desired performance characteristics in the final product. The content of zeolite added to rubber compounds can vary widely depending on the specific requirements of the application and the desired properties of the final rubber product. The optimal concentration of zeolite is determined through a careful balance of factors such as the type of zeolite, its particle size, the rubber matrix, and the intended performance characteristics.

Based on the available scientific literature on the influence of the addition of zeolite on the curing process of rubber compounds, it can be concluded that the content of zeolite in the compound influences the course of the vulcanization reaction [8,11,12,13]. The optimal vulcanization time depends on the amount of zeolite in the rubber matrix [8,11,12,13]. The addition of zeolite to the rubber mixture increases the values of the minimum torque [8,9,11,13,14]. It is assumed that the zeolite in the rubber compound creates an obstacle that hinders the movement of the chains of rubber macromolecules. In the presence of zeolite, physical bonds are created between zeolite and rubber, which also impacts the increase in the minimum torque of the rubber compounds. In the available literature, there is no indication that this effect influences the processing of the rubber compounds; on the contrary, it is considered a positive effect because it delays the very beginning of vulcanization and enables a better distribution of temperatures through the rubber compounds during the vulcanization process. The effect of delaying the start of curing leads to more successful vulcanization and reduces the possibility of degradation of the final product [8,9,11,13,14]. The values of the maximum torque increase with an increase in the amount of zeolite present in the rubber mixture. The increase in the value of the maximum torque is explained not only by the increased degree of curing but also by the impossibility of starting cured polymer chains due to the presence of an increased amount of zeolite. The degree of curing was examined by swelling vulcanized samples in selected solvents, and the results showed that samples with zeolite had a lower degree of swelling than samples without added zeolite [11,12]. Zeolites can act as reinforcing agents in rubber compounds, enhancing mechanical properties [15] such as tensile strength, modulus, and tear resistance. This reinforcement is particularly beneficial in tire manufacturing, where improved durability and performance are essential.

Examination of the morphology by scanning electron microscopy of elastomeric composites confirms that zeolite can be well dispersed in the mass of the rubber compound, but with higher amounts of zeolite, there is a risk of agglomeration, which represents weak points in the structure [8,12,16]. In addition, zeolite leads to an increase in porosity in the mass of the product, which can have a different effect on the final properties depending on the final application [9,17].

This article focuses on studying the impact of adding synthetic Zeolite 4A MS on the curing process and properties of natural rubber-based compounds. The natural rubber-based compounds were mixed with 4A synthetic zeolite at varying proportions (0, 5, 10, 15, 20, and 30 parts per hundred rubbers). Moving die rheometer and isothermal DSC experiments were used to analyze the curing process. The degree of swelling of natural rubber-based composites filled with zeolite in toluene and distilled water was monitored. The morphology of the prepared composites filled with different zeolite content was examined using SEM. Additionally, the tensile strength and hardness were measured using a universal testing machine and durometer Shore A scale, respectively. The thermal stability of the composites filled with different proportions of zeolite was determined by monitoring the sample mass change during programmed heating from room temperature to 550 °C.

## 2. Materials and Methods

### 2.1. Materials

All raw materials used in the work are commercial and were used without additional purification. Natural rubber (NR), commercial name SVR CV60, manufactured by Vietnam Rubber Group, Geruco (Phu Nhuan, Vietnam) was used as the initial precursor. Physicochemical properties of NR are the following: Mooney viscosity at 100 °C 60 ± 5, ash content (max) of 0.40 wt.%, nitrogen content (max) of 0.6 wt.%, dirt content (max) of 0.02 wt.%, and volatile matter of 0.6 wt.%. Cabon black (N330), with an average particle size of 28 to 36 nm, was added to the rubber mixture in a proportion of 50 phr. A semi-efficient vulcanization system was used: a combination of N-isopropyl-N-phenyl-1,4-phenylenediamine (IPPD) and sulfur (S) with the accelerator N-cyclohexyl-2-benzothiazyl-sulfenamide (CBS) and vulcanization activators zinc oxide and stearic acid.

Synthetic zeolite manufactured by Alumina Ltd. (Zvornik, Republika Srpska, Bosnia and Hercegovina), marked 4A MS, was used to examine its influence on the vulcanization process as well as the physical and mechanical properties of the vulcanized elastomeric composite. The symbol 4A suggests that the pore diameter of the zeolite is 4 Å, and the symbol MS indicates that the zeolite can act as a molecular sieve. This zeolite can absorb water and other molecules with a critical diameter of less than 4 Å. The chemical composition of synthetic zeolite 4A MS is shown in Table 1.

### 2.2. Samples Preparation

A series of natural rubber-based compounds filled with varying amounts of synthetic zeolite 4A MS was prepared in a laboratory mixer. Table 2 shows the composition of the prepared rubber mixtures. The proportions of the ingredients of natural rubber composites are expressed in phr. The rubber compounds were mixed in a HAAKE Rheomix laboratory mixer (model 600) with a HAAKE Rheocord EU-5 drive unit. Mixing the rubber compounds was started by masticating natural rubber at a temperature of 90 °C. Table 3 shows the sequence of addition of compounding ingredients and the timing of mixing each cycle at 90 °C. In addition to ensuring a good dispersion of all ingredients in the rubber, care must also be taken to prevent premature vulcanization during mixing, as the process is carried out at 90 °C. That is why the components that participate in the vulcanization reaction were added last, and their mixing time is the shortest, as shown in Table 3.

### 2.3. Characterization

The curing of the rubber compounds was monitored on a moving die rheometer, MDR-A Rotorless Rheometer (manufacturer, Beijing Rade Instrument Co., Ltd., Beijing, China), at a temperature of 150 °C. Isothermal cure experiments are the most common test for control in rubber and elastomer processing. All the essential characteristics, such as minimum and maximum torque (*M_min_* and *M_max_*), scorch times (*t_s_*_2_), and cure times (*t*_90_), are precisely calculated. The curing time, t_90_, is determined from the rheometer curve for the torque value Mt_90_ determined by the equation:

(1) $$Mt_{90}=M_{min}+\left(M_{max}-M_{min}\right)0.9$$

Defining the temperature and time of vulcanization of the rubber mixture is a highly complex task that significantly affects the obtaining of vulcanized products with the desired properties. In industrial conditions, monitoring the changes in the rheological characteristics of the rubber mixture during the chemical reactions of vulcanization usually provides the data needed to guide the curing process in the desired direction. The development and broad application of differential scanning calorimetry (DSC), both in laboratories for fundamental polymer tests and in the production and control process industry, have conditioned its use in monitoring chemical reactions during the curing process. Therefore, in this manuscript, the DSC method was used to monitor the curing process in addition to a rheometer.

The DSC method was used to monitor the curing of the rubber mixture in isothermal conditions at temperatures of 140, 150, 160, and 170 °C for 30 min. A DSC Q20 differential calorimeter (TA Instruments, New Castle, DE, USA) and a hermetic sample pan were used. The mass of the tested samples was about 5 mg. To evaluate the effect of the addition of synthetic zeolite in rubber mixtures, the obtained DSC data were mathematically processed, and the kinetic parameters of the crosslinking reaction were determined.

After additional mixing on a double roller, the rubber compounds were vulcanized in molds in a vulcanizing press. The pressing pressure was set to 150 bar, the vulcanization temperature was 150 °C, and the process lasted 15 min. Then, 2 mm thick blanks were obtained from which samples were cut for testing properties.

The morphological analysis was performed by field emission scanning electron microscopy SEM-EDS (JEOL JSM 6460 LV with EDS device Oxford Instruments INCA; JEOL, Tokyo, Japan) instruments operating at a maximum electron beam of 30 kV.

The chemical structure of composites and zeolite 4A MS was determined using Fourier transform infrared spectroscopy (FTIR). FTIR spectra were recorded on an Thermo Nicolet 5700 FT-IR (Shimadzu, Tokyo, Japan). The instrument has an ATR accessory with a germanium crystal. The spectra were registered at room temperature for wavenumbers between 400 and 4000 cm^−1^ at a nominal resolution of 4 cm^−1^. Each spectrum was acquired with 64 scans.

Toluene and distilled water were used as swelling liquids; the samples were placed in glasses and submerged with sufficient liquid. At defined time intervals, submerged samples were removed from the liquid. Excess liquid was removed from the surface of the samples using filter paper. Measurements were made on an analytical balance with an accuracy of ±0.1 mg (0.0001 g). The weighed samples were then returned to the beaker. From the obtained measurements, the degree of swelling was calculated according to the equation:

(2) $$swelling,\;\%=\frac{m_{2}-m_{1}}{m_{1}}\cdot 100$$

where: *m*_1_—mass before swelling, *m*_2_—after swelling. The better the interactions, the higher the degree of swelling.

Natural rubber-based composites filled with zeolite were tested on the Tensile Tester EZ, Shimadzu (Shimadzu, Tokyo, Japan), according to the ISO 37 standard [18]. The test samples were prepared in a standard Dumbbell shape. The median result of five test pieces is indicated. Hardness testing, according to Shore A, was performed using a durometer, the model manufactured by Sauter. The presented results are averages of five independent measurements on one specimen according to the ISO 7619 standard [19].

A DSC Q20 instrument with a device for controlled cooling down to −90 °C, manufactured by TA Instruments (TA Instruments, New Castle, DE, USA), was used to determine the thermal properties. The test temperature interval is from −90 °C to 150 °C at a heating rate of 10 °C/min. The glass transition temperature values of natural rubber-based composites filled with zeolite were determined from the second heating as the middle of the transition interval.

A TGA Q600 SDT instrument manufactured by TA Instruments (TA Instruments, New Castle, DE, USA) was used to determine the samples’ thermal stability. The prepared natural rubber-based composites filled with zeolite were heated from room temperature to 550 °C, with a heating rate of 5 °C/min in a nitrogen atmosphere.

## 3. Results and Discussion

### 3.1. Curing Characteristics of Natural Rubber-Based Compounds Filled with Zeolite

Table 4 shows the rheological parameters (scorch times (*t_s_*_2_), optimal cure times (*t*_90_), minimum torque (*M_min_*), maximum torque (*M_max_*), and the difference between maximum and minimum torque (Δ*M*)) of the curing reaction determined based on the vulcanization curve of rubber compounds with different amounts of zeolite at 150 °C. The measured torque is proportional to the viscosity of the tested sample, and with an increase in curing, that is, during the vulcanization process, there will be an increase in the value of the torque [18]. The value of *M_min_* of the rubber compounds decreases with the increase in the content of synthetic zeolite, Table 4. For the compounds without zeolite (NR-0phr zeolite), the highest value *M_min_* of 3.18 Nm was read, while for the compounds with the highest proportion of zeolite (NR-30phr zeolite), the lowest value of the minimum torque was 2.67 Nm. The value of *M_min_* depends on the system’s physical filler–filler or filler–rubber interactions before curing. Synthetic zeolite 4A MS has an average particle size of 4 µm, and its addition to the rubber compounds leads to the separation of macromolecular chains of rubber. Thus, their mobility is facilitated, and with an increase in the 4A synthetic zeolite content in the rubber mixture, the viscosity decreases; that is, on the rheometer curves, they get lower minimum torque values [20].

Table 4 shows that increasing amounts of the zeolite in natural rubber-based compounds lead to higher *M_max_* and ∆M values. *M_max_* varies from 10.95 Nm for the rubber mixture without zeolite, while for the compounds in which the parts per hundred rubbers of zeolite are 30, it is 12.67 Nm. Maximum torque is a characteristic of cured rubber, and ∆*M* is a parameter that defines the degree of chemical curing [21,22]. Based on the value of ∆*M*, it can be concluded that adding zeolite to the rubber compounds increased the degree of curing of the system [12,23].

With an increase in the content of zeolite 4A MS in natural rubber-based compounds, there is a slight decrease in the value of the scorch times (*t_s_*_2_), Table 4. Zeolite contains a large number of Si-O groups that have excellent adsorption properties. With an increase in zeolite content in the rubber mixture, there is an accelerated absorption on the surface of silicon dioxide [12]. It can be assumed that there is an interaction between the components of the curing system and the surface of the zeolite particle, and that is why the vulcanization reaction starts earlier with an increase in zeolite in the rubber mixture [13]. On the other hand, the optimal cure time (*t*_90_) lengthens as the zeolite content increases, due to changes in the pH value of the system, which increases the acidity and consequently slows the reaction’s completion [12]. In the literature [24], this result is explained by the fact that the system goes through three stages during vulcanization. In the first stage, filler–filler interactions occur because silicon dioxide has many hydroxyl groups on its surface. In the second stage, accelerated adsorption on the silicon surface occurs. Finally, in the third stage, the speed of curing decreases due to the formation of complex zinc compounds in the interaction between the components of the curing system [24].

**Figure 1:**
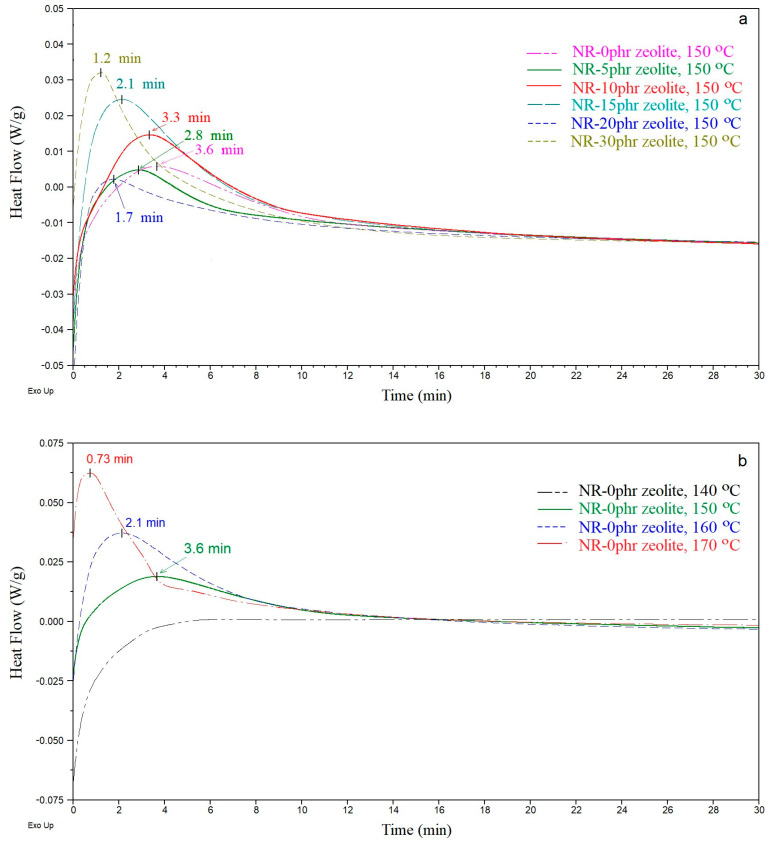
(**a**) Isothermal differential scanning calorimetry thermograms of rubber composites with various amounts of zeolite at 150 °C; (**b**) Rubber compounds without adding zeolite at various temperatures (140, 150, 160, and 170 °C).

Figure 1a shows isothermal DSC thermograms of natural rubber-based compounds filled with different amounts of zeolite at a temperature of 150 °C for 30 min. Exothermic peaks, representing the rubber mixture’s curing process [25], are observed on the DSC curves for all rubber composites at 150 °C. The rate and degree of curing reaction of rubber compounds can be predicted from the peak position and the area under the DSC curves. The amount of added zeolite influences the maximum position of the curing peak. For example, in the sample without the addition of zeolite, the maximum peak is after 3.6 min, while in the sample with 30 phr of zeolite, the maximum is after 1.2 min of curing at a temperature of 150 °C, Figure 1a.

Figure 1b shows the obtained DSC thermograms for the sample without zeolite at various temperatures (140, 150, 160, and 170 °C) for 30 min. The position of the maximum curing peak (the maximum curing speed) shows that it occurs earlier with increased curing temperature (Figure 1b). Based on the DSC curves of the isothermal curing of the natural rubber-based compounds without adding zeolite, it can be concluded that the natural rubber-based compound is not cured at 140 °C for 30 min, Figure 1b.

A comparison among DSC curves of natural rubber-based compounds filled with different amounts of zeolite suggested that the compound containing a higher zeolite loading exhibits a more rapid vulcanization process. This underscores the significant role of zeolite in accelerating the curing reaction of natural rubber-based compounds.

The area under the DSC curve is proportional to the heat change. Δ*H* is the specific (molar) heat of reaction. The reaction rate can be described by the equation:

(3) $$\frac{d\alpha }{dt}=\frac{1}{\Delta H}\left(\frac{dH}{dt}\right)$$

The total heat of reaction Δ*H* is equal to:

(4) $$\Delta H=\int \frac{dH}{dt}dt$$

The partial heat *H* that is released up to a certain moment *t* is related to the degree of conversion (*α*):

(5) α=H/ΔH,

Based on Equation (5), the degree of conversion (curing) with time was determined for all prepared rubber compounds, and the kinetic parameters of the curing reaction were calculated using the general expression for the reaction rate as a function of the degree of reaction:

(6) $$\frac{d\alpha \;}{dt}=k\left(T\right)f\left(\alpha \right),$$

In Equation (6). *t* is time (s), *T* is temperature (K), and *k* is the reaction rate constant. If the curing reaction is assumed to have the nth order of reaction, *f*(*α*) can be written as:

(7) $$f(\alpha )=(1-\alpha {)}^{n},$$

which follows:

(8) $$\log\left(\frac{d\alpha }{dt}\right)=n\;\log(1-\alpha )+\log k,$$

The order of the reaction (*n*) is determined from the slope, and log*k* is the intercept of the resulting line. A linear dependence of log (d*α*/d*t*) on log (1 − *α*) was obtained for all rubber compounds (correlation coefficients were more significant than 0.959). Calculated values for reaction order, energy activation, and rate constant are given in Table 5.

The kinetic parameters were evaluated using non-linear regression analysis, presented in Table 4. The reaction order (n) of curing natural rubber-based compounds has values from 1.28 to 2.10. The values for the reaction rate constant increase with an increase in the curing temperature from 150 °C to 160 °C, while it can be observed that for all samples at a temperature of 170 °C, the values of the reaction rate constant decrease. A temperature of 170 °C is a relatively high temperature, and it can be assumed that in addition to the curing reaction, degradation of the sample also occurs, which can explain the decrease in the reaction rate constant. When the maximum rate of curing occurs quickly, it becomes difficult to control the curing process. Therefore, choosing a temperature of 150 °C for vulcanization is optimal. Fifteen minutes was selected for the pressing time because the curing peak was completed after that time.

### 3.2. Morphology of Natural Rubber-Based Composites Filled with Zeolite

**Figure 2:**
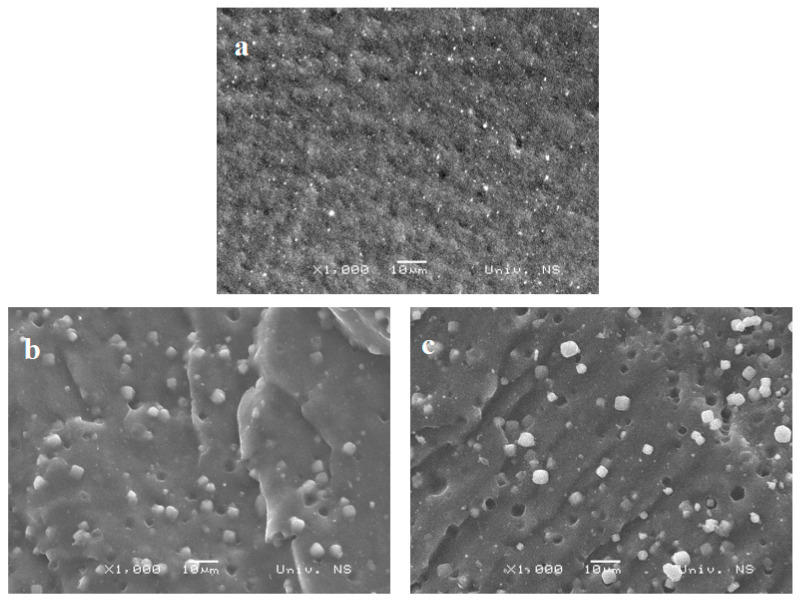
SEM images of a vulcanized composite based on natural rubber: (**a**) without zeolite, (**b**) with 20 phr zeolite, and (**c**) with 30 phr zeolite.

The morphology of natural rubber-based composites prepared and filled with different zeolite content was determined using SEM. Figure 2 shows SEM images of samples without zeolite and composites with 20 and 30 phr zeolite 4A MS at a magnification of 1000 times. Based on the SEM images, it can be seen that the zeolite particles are well dispersed in the composite. Even with high contents of zeolite 4A MS (20 and 30 phr) in the composite, no agglomerates were formed. However, with an increase in the zeolite content in the NR matrix, the porosity of the examined composites increases, which may impact the final properties [9,17].

**Figure 3:**
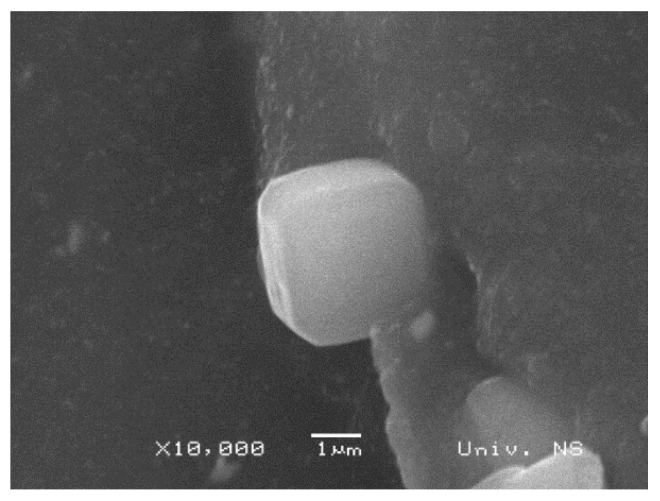
SEM image of a particle of synthetic zeolite 4A MS.

**Figure 4:**
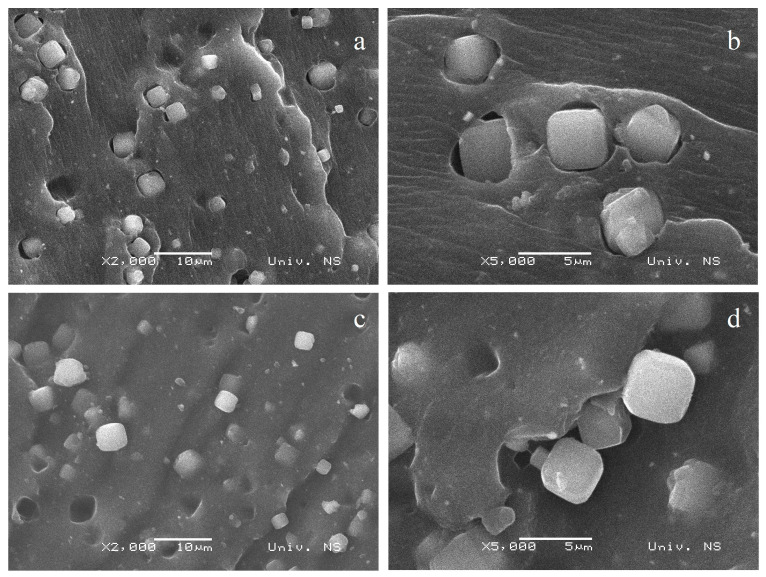
SEM images of natural rubber-based composites filled with 20 phr (**a**,**b**) and 30 phr zeolite (**c**,**d**).

A closer look at a synthetic zeolite 4A MS particle embedded in the composite is shown in Figure 3. A cube-shaped particle of about 4 µm can be seen. Zeolite’s compatibility with natural rubber was investigated in more detail and presented in Figure 4. Based on the SEM images shown in Figure 4, it can be seen that there are pores between the zeolite particles and the NR matrix, and pores of different sizes can be observed. Pores represent points of zero strength. The physical properties of the composite deteriorate due to poor contact of the filler with rubber, so it can be assumed that with significant contents of zeolite 4A MS, the mechanical properties of the composite will deteriorate.

### 3.3. FTIR Spectrum Analysis of Natural Rubber-Based Composites Filled with Zeolite

**Figure 5:**
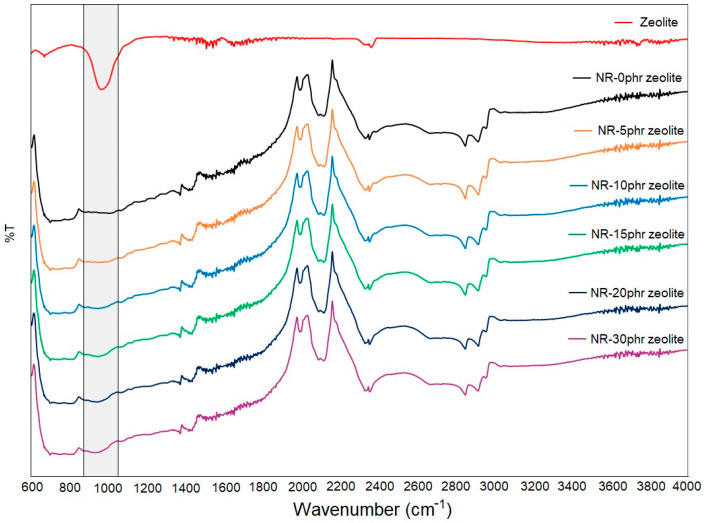
FTIR spectra of composites based on natural rubber with 0, 5, 10, 15, 20, and 30 phr zeolite and the FTIR spectrum of pure zeolite 4A MS.

Figure 5 shows the comparative FTIR spectra of composites based on natural rubber with 0, 5, 10, 15, 20, and 30 phr zeolite, as well as the FTIR spectrum of pure zeolite used in this work.

The FTIR spectrum provides information on the molecular structure and functional groups present in the composite. Natural rubber primarily consists of poly (cis-1,4-isoprene) chains, so the FTIR spectrum shows peaks corresponding to the stretching of these groups. In the 2536–2996 cm^−1^ range, the peaks that appear are due to symmetric and asymmetric stretching vibrations of the –CH_3_ bond present in aliphatic macromolecular chains [26]. The peak occurring at 2159 cm^−1^ represents the vibration of the –C≡C– triple bond in carbon black. The band at 1466 cm^−1^ is due to the stretching of the –CH_2_– bond. Peaks occurring at 1381 cm^−1^ are due to the stretching of the –CH bond in the methyl groups of the macromolecular chain [26]. In the FTIR spectrum of the zeolite, bands located at 968 and 670 cm^−1^ are due to the external vibration of (Si, Al)–O asymmetric stretching and internal vibration of (Si, Al)–O symmetric stretching, respectively [27]. The peak at 465 cm^−1^ is assigned to the internal vibration of (Si, Al)–O bending observed [28]. The bands at 1668 and 3565 cm^−1^ due to vibration OH group are also observed.

If the FTIR spectra of composites with different zeolite content are compared, it can be seen that the only noticeable change is in the 900–1100 cm^−1^ region, Figure 5. With an increase in the proportion of zeolite in the composite, the intensity of the peak in this region becomes more intense. This is expected due to the growth of the zeolite content in the system because the strong band in the area of 900–1100 cm^−1^ corresponds to the stretching vibrations of the (Si, Al)–O bonds in the zeolite framework.

### 3.4. Degree of Swelling of Natural Rubber-Based Composites Filled with Zeolite

**Figure 6:**
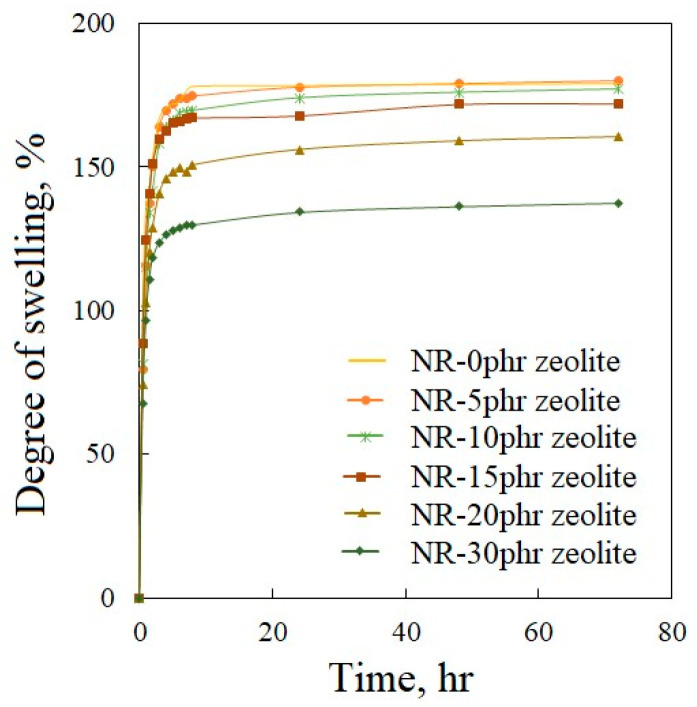
Dependence of the degree of swelling of vulcanized natural rubber-based composites filled with zeolite on the time immersed in toluene.

Figure 6 shows that the degree of swelling depends on how long the sample is immersed in toluene. More significant changes in the degree of swelling are observed in the first three hours. Based on the obtained values of the degree of swelling, it can be seen that the swelling is most dominant in the first half hour. After half an hour in toluene, all samples swelled by more than 67%. After 5 h in toluene, the equilibrium degree of swelling is reached, after which there are no more significant changes in the mass or dimensional changes in the samples. The addition of zeolite to rubber mixtures affects the degree of swelling. The sample without zeolite after 30 min in toluene swelled by 79.6%, while the sample with 30 phr of zeolite after 30 min had a swelling degree of 67.6%. The sample NR-30phr zeolite has the lowest maximum swelling degree (137.6%), while the swelling degree of the sample without zeolite after 72 h is 180.1%.

Zeolites are known for their high absorptive capacity due to their microporous structure. They can absorb liquids, which might reduce the overall swelling of the composites as some of the liquid is taken up by the zeolite particles instead of the rubber matrix. However, if zeolite particles are well-dispersed and interact effectively with the rubber chains, they can restrict chain mobility. The physical and chemical interaction between the zeolite and the rubber matrix can enhance the crosslink structure, reducing the extent of swelling. Additionally, zeolite particles can block or obstruct the pathways that swelling agents typically take to penetrate the rubber matrix, further reducing swelling. In general, the appropriate amount of zeolite positively affects the curing of natural rubber-based compounds [13]. Based on the degree of swelling, it can be concluded that the degree of curing is higher in the sample containing a higher proportion of zeolite. Reduced swelling can improve the material’s dimensional stability and mechanical properties.

**Figure 7:**
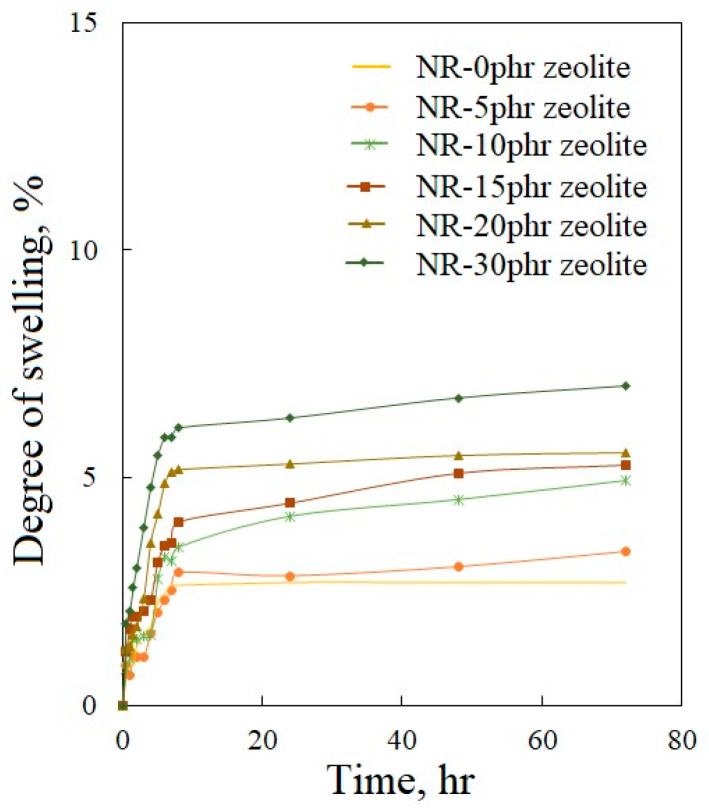
Dependence of the degree of swelling of vulcanized natural rubber-based composites filled with zeolite on the time immersed in distilled water.

Measuring the swelling of cured composites in distilled water does not provide information about the curing of the system, but these data can indicate the degree of porosity of the examined composites. Figure 7 shows that there are no significant changes in the mass of the samples in distilled water. Swelling is slightly more in samples containing larger amounts of zeolite. This result is expected because zeolite is a microporous material, and it is precisely this microporous structure that makes it a material that is particularly significant for absorption and can contribute to greater water absorption in the composites under the study. Also, the result that there was a greater swelling in distilled water in samples containing larger amounts of synthetic zeolite is another confirmation that with an increase in the amount of zeolite in the composites, there is a greater porosity in the structure of the tested composites.

The swelling behavior of a tested sample in water compared to toluene was significantly different due to the distinct nature of these liquids and their interaction with both the rubber matrix and the zeolite particles. The surface properties of the zeolite, including its hydrophilicity or hydrophobicity, can influence the interaction with the natural rubber and the swelling medium. Toluene is a nonpolar, hydrophobic solvent that can interact more strongly with the hydrophobic parts of the natural rubber matrix, leading to greater swelling. Zeolites are less effective at absorbing nonpolar solvents like toluene than water, so toluene is more likely to penetrate the natural rubber matrix and cause swelling. Zeolites are generally hydrophilic and can absorb water into their porous structure. Since water is a polar solvent, its interaction with the hydrophobic parts of the natural rubber is limited, further reducing swelling. The porous structure can absorb and retain more water within the voids.

### 3.5. Mechanical Properties of Natural Rubber-Based Composites Filled with Zeolite

The test data in Table 6 shows that with a small addition of zeolite (up to 10 phr), there is no significant change in the tensile strength values. Adding a considerable amount of zeolite to the natural rubber matrix results in a reduction of the tensile strength values. Phatcharasit et al. showed that with an increase in the zeolite content above 10 phr in the rubber mixture, mechanical properties deteriorate due to the inability of the filler to withstand stresses [6,10]. The dispersion of the filler in the rubber matrix was assumed to be poor, leading to agglomeration in the system [29,30]. Bukit et al. concluded that the samples filled with zeolite have a higher tensile strength than those without zeolite, which implies that the addition of zeolite led to an increased ability of the samples to withstand tensile stress [10]. This ability is in accordance with the nature of zeolite as a rigid material; therefore, the increased stiffness of the composite is expected. However, adding a more significant amount of zeolite leads to the creation of an interphase zeolite/NR matrix, representing weak points in the product [10]. Based on the results of the SEM analysis and the degree of swelling of the examined composites in distilled water, it was concluded that the system’s porosity increases with the increase in the proportion of zeolite in the rubber. So, with large proportions of zeolite 4A MS in the composite, the mechanical properties deteriorated due to an increase in the porosity of the composite. Therefore, it was assumed that the composite’s tensile strength values are reduced further with a higher zeolite content.

Elongation at break decreased with increasing zeolite content from 1790% for the sample without zeolite (NR-0phr zeolite) to 1272% for the sample with 30 phr zeolite (NR-30phr zeolite), which is a reduction in elongation at break of almost 30%. This could be due to the increased degree of curing in the composites as the proportion of zeolite in the natural rubber matrix increases [8]. The samples’ elongation values decreased due to increased rubber composites’ crosslink density [26].

The modulus of elasticity is used to characterize the composites’ stiffness and degree of curing. Tensile moduli represent the force per unit area of the test tube at a given elongation of 100% and 300%. The higher the actual stress at a given elongation, the greater the rigidity of the cross-linked material. With the increase in the share of zeolite in natural rubber-based composites, the values of the modulus of elasticity increase. The sample without zeolite (NR-0phr zeolite) requires 2.28 MPa to achieve 100% elongation, whereas the composite with 30 phr of zeolite requires 4.91 MPa. The modulus of elasticity at 300% elongation increased from 6.16 MPa to 8.49 MPa by adding 30 phr of zeolite to the NR matrix. Adding a small amount of zeolite in the rubber mixture increases the value of tensile strength and modulus of elasticity compared to the sample without zeolite, which is in accordance with the literature data [11,12,31].

Table 7 gives the hardness measurement results according to the Shore A method. The hardness values of all samples are in the application range of medium-hard and hard rubber materials (50–90 ShA) [21]. It can be observed that the hardness of the composite increases with the increase in the proportion of zeolite in the rubber mixtures.

It follows from the literature that the hardness values increase with an increase in the proportion of zeolite up to the optimal amount. These results are expected because zeolite is a ceramic material with higher hardness than elastomeric materials. However, after exceeding the optimal mass fraction of zeolite in the rubber mixture, an interphase is formed, and then more addition of zeolite does not have a strengthening effect in elastomeric composites [8,11,14,31]. There are also exceptions, namely in the paper [14,32], where it was observed that the hardness of the examined composites decreases even with a small addition of zeolite below 1 phr. It was explained that this is a consequence of using additional retardants in the recipe for the rubber mixture [14,32].

### 3.6. Thermal Properties of Natural Rubber-Based Composites Filled with Zeolite

**Figure 8:**
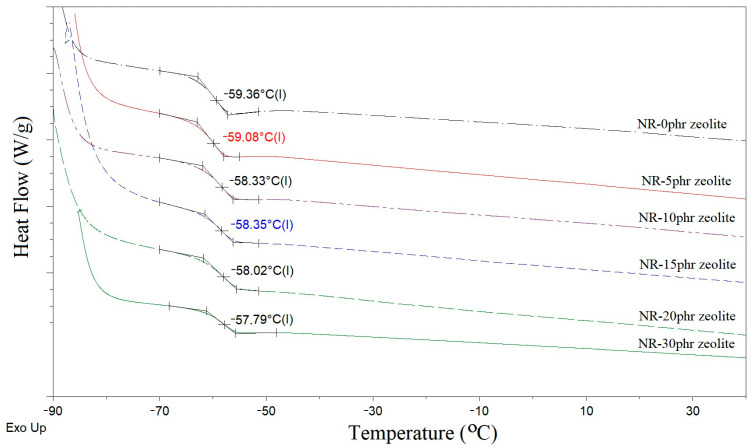
DSC curves of natural rubber-based composites filled with different proportions of zeolite.

Figure 8 shows the DSC curves of heat flow of natural rubber-based composites filled with zeolite from −90 to 40 °C at a heating rate of 10 °C/min. The glass transition temperature of natural rubber-based composites increases slightly with an increase in the proportion of zeolite. Based on the data shown in Figure 8, it can be concluded that zeolite slightly affects the mobility of macromolecular chains in natural rubber. In the available works examining zeolite as fillers in elastomeric composites, it was not observed that the addition of zeolite significantly or at all affects the glass transition temperature [17]. Adding a more significant amount of zeolite to the system (20 or 30 phr) moved the transition from the glassy state to slightly higher temperatures (from −59.36 °C to −57,79 °C; Figure 8). It can be assumed that molecular mobility is reduced due to the formation of bonds between the macromolecule chain and zeolite.

### 3.7. Thermal Stability of Natural Rubber-Based Composites Filled with Zeolite

**Figure 9:**
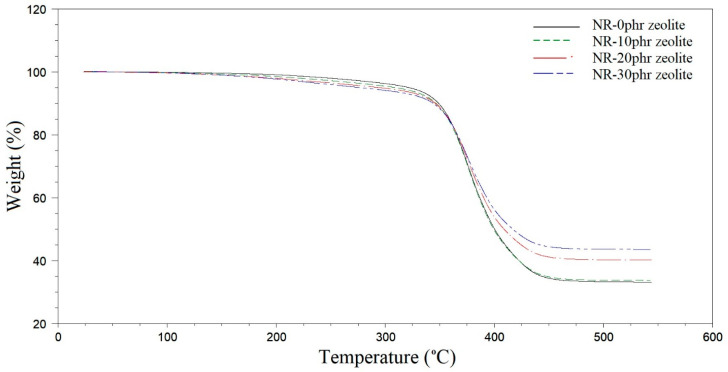
Thermal gravimetric analysis (TGA) of natural rubber-based composites filled with zeolite.

The curves of mass change with temperature are shown in Figure 9. The tested samples had 0, 10, 20, and 30 phr of zeolite. Based on the obtained TG curves, it can be observed that the amount of added zeolite affects the initial stages of thermal decomposition of the examined samples and the rest after the analysis at a temperature of 500 °C.

The amount of added zeolite affects the beginning of the thermal decomposition of the tested samples (up to 10% mass degradation). As the zeolite content in the composite increases, the temperature at which 5% of the sample mass is degraded decreases. In the sample without zeolite, a 5% mass loss was achieved at a temperature of 322 °C, while in the sample with 30 phr zeolite, a mass loss of 5% was achieved at a temperature of 277 °C, Table 8. After the degradation of 10% of the sample, there is a uniform trend of further thermal degradation of samples with different zeolite content.

It was expected that zeolites could improve thermal stability by acting as a barrier to heat and mass transfer, delaying the decomposition of composites. However, the porous structure of a natural rubber-based composite filled with a significant proportion of zeolite can significantly influence the initial stages of thermal decomposition as determined by TG analysis. The presence of pores can serve as sites for the initiation of decomposition. In a porous structure, the decomposition products (gases) can escape more readily, which may affect the kinetics of the decomposition process. Additionally, zeolites possess catalytic properties due to their active sites, which can lower the activation energy for decomposition reactions. This catalytic activity can result in an earlier onset of thermal degradation. In addition, residual water in the pores of the zeolite, which can also contribute to the initial reduction in mass of the composites, cannot be excluded entirely.

The residue after TG analysis increases with the increase in zeolite proportion. At a temperature of 500 °C, in the sample without zeolite, 66.8% of the sample mass was degraded, while in the sample containing 30 phr of zeolite, 56% of the sample mass was degraded. Zeolite is an inorganic material and does not decompose in the temperature range in which the natural rubber part of the composite decomposes, so larger masses of ash were obtained with an increase in the proportion of zeolite in the system [10,13,16,32].

## 4. Conclusions

Synthetic zeolite 4A MS has an average particle size of 4 µm, and its addition to the rubber compounds leads to the separation of macromolecular chains of natural rubber. Thus, their mobility is facilitated, and the viscosity decreases with increased 4A synthetic zeolite content in the rubber mixture. The rheological parameters were determined to investigate the impact of 4A synthetic zeolite on the curing process. Based on the values of M*max* and ∆M, it can be concluded that adding zeolite to the rubber compounds increased the degree of curing of the system. It can be assumed that there is an interaction between the components of the curing system and the surface of the zeolite particle, and that is why the vulcanization reaction starts earlier with an increase in zeolite in the rubber mixture. A comparison of DSC curves suggested that the compound containing a higher zeolite loading exhibits a more rapid vulcanization process. This underscores the significant role of zeolite in accelerating the curing reaction of natural rubber-based compounds. Adding a considerable amount of zeolite (above 15 phr) to the natural rubber matrix deteriorates the tested mechanical properties. Based on the results of the SEM analysis and the degree of swelling of the examined composites in distilled water, it was concluded that the system’s porosity increases with the increase in the proportion of zeolite in the rubber. It can be assumed that with large proportions of zeolite 4A MS in the composites, the mechanical properties deteriorated due to increased porosity. The hardness values of all samples are in the application range of medium-hard and hard rubber materials (50–90 ShA). Based on the obtained TG curves, it can be observed that the amount of added zeolite affects the initial stages of thermal decomposition of the examined samples and the rest after the analysis at a temperature of 500 °C. The presence of pores can serve as sites for the initiation of decomposition. In a porous structure, the decomposition products (gases) can escape more readily, which may affect the kinetics of the decomposition process. Zeolite is an inorganic material and does not decompose in the temperature range in which the natural rubber part of the composite decomposes, so larger masses of ash were obtained with an increase in the proportion of zeolite in the system.

## Figures and Tables

**Table 1:** Chemical composition of synthetic zeolite 4A shown as weight percentage (unit %).

	Typical Content
Water content, 950 °C, 2 h	19%
Al_2_O_3_	36%
Na_2_O	22%
SiO_2_	42%

**Table 2:** Ingredients in natural rubber-based compounds filled with synthetic zeolite 4A MS (phr—parts per hundred rubber).

	NR-0phr Zeolite	NR-5phr Zeolite	NR-10phr Zeolite	NR-15phr Zeolite	NR-20phr Zeolite	NR-30phr Zeolite
Natural rubber, (phr)	100	100	100	100	100	100
Carbon black, (phr)	50	50	50	50	50	50
**Zeolite**, (phr)	0	5	10	15	20	30
Zinc oxide, (phr)	4	4	4	4	4	4
Stearin, (phr)	1	1	1	1	1	1
IPPD, (phr)	1	1	1	1	1	1
Sulfur, (phr)	2.5	2.5	2.5	2.5	2.5	2.5
CBS, (phr)	0.5	0.5	0.5	0.5	0.5	0.5

**Table 3:** The sequence of addition of ingredients and the time of mixing cycle at 90 °C.

Ingredients	The Time of the Mixing Cycle, (min)	Revolutions per Minute, (rpm)
Mastication of natural rubber	6	100
Added ingredients in the presence of which the vulcanization process will not start (carbon black, zeolite, zinc oxide, stearin, IPPD)	5	60
Added a vulcanization system (sulfur, CBS)	2	60

**Table 4:** Parameters of the vulcanization reaction at 150 °C determined based on the vulcanization curves of natural rubber-based compounds filled with zeolite.

Designation	Minimum Torque, *M_min_* (Nm)	Maximum Torque, *M_max_* (Nm)	Δ*M* (Nm)	Scorch Time, *t_s_*_2_ (min:s)	Optimum Cure Time, *t*_90_ (min:s)
NR-0phr zeolite	3.18	10.45	7.27	1:20	7:50
NR-5phr zeolite	3.14	10.49	7.35	1:19	8:16
NR-10phr zeolite	3.10	10.52	7.42	1:19	8:31
NR-15phr zeolite	3.05	10.95	7.90	1:17	8:41
NR-20phr zeolite	3.02	11.18	8.16	1:16	8:47
NR-30phr zeolite	2.67	12.67	10.00	1:09	9:06

**Table 5:** Kinetic parameters of the curing reaction of rubber compounds: reaction rate constant (*k*), reaction order (*n*), correlation coefficient (*R*^2^), and energy activation (*Ea*).

Designation	Temperature, (°C)	*k*, (s^−1^)	*n*	*R* ^2^	*Ea*, (kJ/mol)
NR-0phr zeolite	150	0.002194	1.73	0.9924	64.3
	160	0.004099	1.47	0.9944	
	170	0.002414	2.10	0.9961	
NR-10phr zeolite	150	0.001887	1.69	0.9847	65.8
	160	0.002753	1.76	0.9914	
	170	0.001186	1.28	0.9587	
NR-20phr zeolite	150	0.002226	1.81	0.9938	63.6
	160	0.003272	1.63	0.9949	
	170	0.005111	1.36	0.9898	

**Table 6:** Mechanical properties of vulcanized natural rubber-based composites filled with zeolite.

	NR-0phr Zeolite	NR-5phr Zeolite	NR-10phr Zeolite	NR-15phr Zeolite	NR-20phr Zeolite	NR-30phr Zeolite
Tensile stress, (N)	325.4 ± 17.1	320.6 ± 15.4	317.4 ± 14.1	255.3 ± 13.4	259.8 ± 13.6	221.2 ± 12.8
Tensile strength, (MPa)	25.8 ± 1.4	25.7 ± 1.2	26.5 ± 1.8	21.3 ± 1.1	20.6 ± 1.1	18.4 ± 1.1
Elongation at break, (%)	1790 ± 94	1674.5 ± 80	1523 ± 78	1497 ± 78	1373 ± 72	1272 ± 74
Modulus of elasticity at 100% elongation, (MPa)	2.28 ± 0.1	2.94 ± 0.14	3.13 ± 0.14	3.54 ± 0.19	4.15 ± 0.22	4.91 ± 0.28
Modulus of elasticity at 300% elongation, (MPa)	6.16 ± 0.32	6.9 ± 0.33	7.31 ± 0.32	8.10 ± 0.42	8.31 ± 0.43	8.49 ± 0.49

**Table 7:** Shore hardness (shore A) natural rubber-based composites filled with zeolite.

	NR-0phr Zeolite	NR-5phr Zeolite	NR-10phr Zeolite	NR-15phr Zeolite	NR-20phr Zeolite	NR-30phr Zeolite
Shore hardness, (ShA)	66 ± 0.6	67.5 ± 0.5	68.5 ± 0.5	70.5 ± 0.4	72.5 ± 0.5	75.5 ± 0.6

**Table 8:** Thermal stability of natural rubber-based composites filled with zeolite.

	NR-0phr Zeolite	NR-10phr Zeolite	NR-20phr Zeolite	NR-30phr Zeolite
Temperature at 5% of weight loss, (°C)	322	310	295	277
Temperature at 10% of weight loss, (°C)	349	346	346	344
Temperature at 20% of weight loss, (°C)	366	365	366	366
Temperature at 30% of weight loss, (°C) Temperature at 40% of weight loss, (°C) Temperature at 50% of weight loss, (°C)	376	376	378	379
	387	386	390	393
	400	399	409	417
Weight loss at 500 °C, (%)	66.8	66	59.7	56

## Data Availability

The original contributions presented in the study are included in the article, further inquiries can be directed to the corresponding author.
